# Supplementary material for: Eye-Gaze direction triggers a more specific attentional orienting compared to arrows
Source: PLoS One. 2023 Jan 25;18(1):e0280955. doi: 10.1371/journal.pone.0280955 (PMC9876282; doi:10.1371/journal.pone.0280955)
Supplement: Attachment — Submitted filename: Plos_Peer Review.docx [file pone.0280955.s001.docx]

Peer Review

6. Review Comments to the Author

**Introduction:**

The comparison of biologically/socially relevant attentional cues (e.g eye-gaze, pointed hands, head orientation and body orientation) versus non-social cues ( e.g arrows) has a rich history in the field of attention and more generally social cognition and perception. The introduction would benefit from a more in-depth description of some of the research in the field. Below are some references to relevant papers that the authors may wish to include. These are papers that have compared attentional orienting to social vs. non-social directional cues as well as papers that have examined perceptual representation of these directional cues. And more broadly, paper that have discuss how individual differences may account for the results we see in social attention with respect to cue type.

- Capozzi, F., & Ristic, J. (2018). How attention gates social interactions. *Annals of the New York Academy of Sciences*, *1426*(1), 179-198.
- Dalmaso, M., Castelli, L., & Galfano, G. (2020). Social modulators of gaze-mediated orienting of attention: A review. *Psychonomic Bulletin & Review*, *27*(5), 833-855.
- Guzzon, D., Brignani, D., Miniussi, C., & Marzi, C. A. (2010). Orienting of attention with eye and arrow cues and the effect of overtraining. *Acta Psychologica*, *134*(3), 353-362.
- Cooney SM, O’Shea A, Brady N (2015) Point Me in the Right Direction: Same and Cross Category Visual Aftereffects to Directional Cues. PLoS ONE 10(10): e0141411.

**Sample size, Composition & Statistical Power**

How was the sample size estimated for the three experiments? 37 experiment 1,75 experiment 2, 26 for experiment 3. Justification for sample size needs to be given.

The majority of the sample in all three experiments is female. Please comment on this as a limitation with reference to previous research that has identified sex differences in spatial orienting of attention in similar Posner cueing experiments know differences in how males and female orient their attention (see Cooney, Brady & Ryan 2017; Bayliss, di Pellegrino & Tipper, 2005, Mitsuda, T., Otani, M., & Sugimoto, S. (2019).

Exp 3 is a replication but with less than half the sample of experiment. The author’s then go on to run a combined analysis for experiment 2 & 3. Further justification is required here. Presumably, the combined analysis was conducted because Exp 3 contained a very small sample size. However, it’s important to note that the participants in Exp 3 only took part in one condition. While the authors mention this choice they do not justify it – why did the participants only do the placeholder present condition? The authors need to justify this choice in relation to the power of the sample in Exp 3. In general, the power and justification for sample size in all 3 experiments requires justification and discussion.

I have reservations about the combined analysis as it does not follow best research practice. Given that the result of the cue-type ( *arrow, eye gaze*) * cue-target relation (*same-location/same-hemifield vs. opposite-location/opposite- hemifield*) changes (i.e. it becomes statistically significant) when the sample size is increased in the combined analysis and the study does not seem to be pre-registered, I recommend removing the combined analysis. Or at the very least detailing the choice and the possible limitations of this approach.

Method: for the arrow trials a horizontal line was placed…presumable this was a horizontal line with an arrowhead – this need to be specified.

SOA: Would the authors expect different results if the SOA was manipulated?

Why was 300ms chosen?

Instead of describing three studies refer to each as Experiment 1, Experiment 2, Experiment 3.

Exp 2 and 3 are online experiments whereas Exp 1 was in-person. As Exp 2 & 3 were online, considerably less control over the way the stimuli were viewed., i.e did all participant take part on laptopc/Pc’s?

*Post-hocs* should be reported for significant interactions. The authors refer to figures to make inferences about the interactions – at the very least the *post- hocs* should be in the supplementary.

Experiment 1 - Method

Please report the size and dimensions of the computer screen/monitor.

How was ‘unpredictability’ of the target X and O operationalized? Was there an equal amount of trials for X and O targets?
