# Supplementary material for: Eye-Gaze direction triggers a more specific attentional orienting compared to arrows
Source: PLoS One. 2023 Jan 25;18(1):e0280955. doi: 10.1371/journal.pone.0280955 (PMC9876282; doi:10.1371/journal.pone.0280955)
Supplement: Attachment — Submitted filename: Response to Reviewers.docx [file pone.0280955.s002.docx]

June, 2022

Dear Academic Editor,

I hereby enclose the revised version of manuscript number PONE-D-22-03696R1 titled *“Eye-Gaze direction triggers a more specific attentional orienting compared to arrows”.*

We very much appreciate your comments as well as the reviewers’ suggestions; we have tried to address all of them in this new version of the manuscript. We agree that the points raised by you and by the reviewers have contributed to strengthening the document. Below we detail the changes in the manuscript in response to the reviewers’ comments.

We hope that after these final changes the manuscript is suitable for publication. Furthermore, we are willing to make any new change if requested.

Thank you again for the opportunity, and for your time and consideration.

Sincerely,

Jeanette A. Chacón-Candia (on behalf of the other authors)

Departamento de Psicología Experimental

Campus de Cartuja, s/n

18071 Granada – Spain

jeanettealicia.chaconcandia@uniroma1.it / chaconcandia@correo.ugr.es

**Reviewer #1**

- I was a little confused by some of your statistical analyses. It sounds like you did separate analyses for general cueing effects and hemifield effects, for each of the placeholder-present and placeholder-absent conditions. Why were these conditions analyzed separately?. It seems like the conclusion that shifts of attention to gaze and arrow cues differ in the specificity of their attentional focus (which is modulated by the placeholders) necessitates directly testing these interactions. For example, in Experiment1, you really have 4 cue-target relations (same location, opposite location, same hemifield, opposite hemifield), 2 cue types (gaze, arrow), and 2 placeholder conditions (present, absent), which could/should be analyzed in one ANOVA. There are some interesting patterns in the data that are not being addressed by analyzing these separately (e.g., in Exp. 2, for gaze cues the opposite-location/opposite-group RTs are faster than both same group and opposite group, and for arrow cues the opposite location is faster than the opposite group, both of which are unexpected).

*Response:* Thank you for your comments. The analyses you suggested are now reported in the revised version of the paper (See Experiment 1 & 2, Results section).

- It appears that eye movements were not monitored (understandable, given the shift to online data collection), but is it possible that there are differences in eye movements to the two cue types that could explain the patterns of results? Do gaze cues elicit more overt orienting, which could account for the greater specificity of the attentional allocation?

*Response:* Several studies have suggested that there are no differences between the overt attention produced by eye-gaze and arrow stimuli (Khun & Benson, 2007; Khun et al., 2010; Khun & Kingstone, 2009). However, even if such a difference between the two cue types does exist, we would expect it to affect in the same manner in both, the placeholder-present and the placeholder-absent conditions. Nevertheless, our patter of results shows a difference between the two stimuli, but only when placeholder objects are presented in the scene, suggesting that the observed differences are not due to the over attention produced by eye-gaze and arrows, but may be due to the distinct ways in which people select objects in response to them.

References

Kuhn, G., & Benson, V. (2007). The influence of eye-gaze and arrow pointing distractor cues on voluntary eye movements. *Perception & psychophysics*, *69*(6), 966-971.

Kuhn, G., Benson, V., Fletcher-Watson, S., Kovshoff, H., McCormick, C. A., Kirkby, J., & Leekam, S. R. (2010). Eye movements affirm: automatic overt gaze and arrow cueing for typical adults and adults with autism spectrum disorder. *Experimental Brain Research*, *201*(2), 155-165.

Kuhn, G., & Kingstone, A. (2009). Look away! Eyes and arrows engage oculomotor responses automatically. *Attention, Perception, & Psychophysics*, *71*(2), 314-327.

- I have a minor concern about the cues themselves and how that might affect the observed pattern. For the arrow cues, the horizontal line is present and then the arrowhead appears at cue onset, whereas for the gaze cues the pupils are present and then move to the left or the right at cue onset (which may be programmed as the appearance of a new object, but I imagine to the participant is perceived as the movement of the pupils). How does appearance of a new object vs. movement of an existing object affect cueing and the orienting of attention? I.e., is the difference observed really between gaze and arrows, or is it because of lower-level differences in object processing?

*Response:* This is an interesting point that we have now commented on as a limitation in the revised version of the paper (see, General discussion section). Nonetheless, if this phenomenon influenced orienting of attention, the same influence should be observed in the placeholder-absent condition. However, no gaze-arrow differences exist when no placeholders are presented on the scene.

- The final paragraph at the end of the conclusion either needs to be removed or expanded to include citations and a more thorough discussion of why understanding different mechanisms for gaze and arrow cues is important. Right now, it is too vague and doesn’t add anything to the paper.

*Response:* Thank you for the observation. This point has been re-address in the revised version of the paper.

- *Although not a major concern, to fully understand the differences between these tasks it might be useful to dig into the data in Exp. 2 and 3 further:*

1) Were there distance effects in Exp. 2 or 3? In both these experiments the cued/opposite placeholder is on the horizontal meridian and then the two within-group placeholders are at a graded distance above or below this location. It appears that both locations within the group were combined together for analysis, but one would imagine a differential “spread” of attention to nearby and more distant placeholders. It might be useful to directly assess this.

*Response:* Thank you for your suggestion; this is an interesting issue. Although we did not include this analysis in the revised version of the paper, we explored, as you suggested, the data related to the cue-target distance when the placeholder-present grouping effect was to be analysed.

By doing this analysis in experiment 2, we indeed found an effect of distance (near vs fare; F_1,74_=36.76, *p*=<.001,η^2^p=0.332), showing that, in general, reaction times were faster when targets appeared at the grouping-near location than on the grouping-far location. The interaction cue-type X validity X distance (F_1,74_=5.38, *p*=.023,η^2^p=0.068) was also significant, revealing that when arrows were used as a cue, beside a main effect of validity (F_1,74_=13.09, *p*=<.001,η^2^p=0.150; lower reaction times for valid [M=559, SD=80.34] than for invalid [M=571, SD=82.13] trials), participants responded faster when the target appeared at the near than at the fare location in both the valid (M=548 SD=76.24 vs M=570 SD=88.86) and invalid trials (M=566 SD=85.27 vs M=575 SD=85.25). Importantly, no interaction of validity X distance was found when analysing the arrow-grouping effect (*p*>.05). When eye gaze was used, the distance effect was also significant (F_1,74_=19.16, *p*=<.001,η^2^p=0.206), showing again that participants responded faster when the target appeared at the nearest than at the furthest location in both the valid (M=558 SD=82.73 vs M=569 SD=80.40) and invalid trials (M=556 SD=72.97 vs M=575 SD=82.73). However, nor the validity nor the interaction of validity X distance were significant (*p*>.05).

Experiment 3 has been removed in the revised version of the paper, which is the reason why we are not reporting the suggested analysis from that experiment.

2) Were there differences between visual fields (upper/lower and left/right)? When one group is in the lower VF, the opposite group is always in upper VF (and vice versa). There might be asymmetries in how attention is allocated (i.e., easier to shift attention downward than upward, might see less of a group effect when attention is directed upward and then more easily shifts downward on invalidly cued trials). Similarly, you might see effects of left vs. right (easier to shift attention from left to right), or an interaction between left/right and up/down, such that effects when cued group is in upper left will be very different when it is in lower right.

*Response:* Thank you for your suggestion; this is an interesting issue. Although we did not include this analysis in the revised version of the paper, as it was not the main goal of the study, we explored, as you suggested, the data related to the hemifields up/down and left/right.

First, the analysis reported a main effect of hemifield (up vs down) when an arrow was presented as a cue (F_1,74_=8.94, *p*=.004,η^2^p=0.108), showing that in this placeholder-present condition, participants responded faster when targets appeared at the upper than at the lower hemifield in both valid (M=567, SD=91.72 vs M=573, SD=92.74) and invalid trials (M=566, SD=82.67 vs M=584, SD=94.37). When eye gaze was used, no up/down hemifield effect was found (*p*>.05).

Secondly, when analysing the left/right hemifield effect, no hemifield or any related interactions were found (all *p*>.05).

Experiment 3 has been removed in the revised version of the paper, which is the reason why we are not reporting the suggested analysis from that experiment.

- There are some typographical and grammatical errors throughout the manuscript. These are generally minor and don’t impede understanding of the paper, but a thorough proof-reading is recommended.

*Response:* Thank you for the observation. The signalled errors have been corrected in the revised version of the paper. We also have reviewed the entire manuscript again to try to point out and correct any other possible errors.

**Reviewer #2**

The study includes three experiments that examined whether the inclusion of a placeholder would modulate orienting of attention in a Posner cueing paradigm to socially relevant versus socially non –relevant directional cues. Experiment 2 and 3 were conducted online and Experiment 1 was an in-person laboratory experiment. Results support previous findings that have shown that when an object is present eye-gaze cueing facilitates attention to object part whereas arrows to the entire object.

Overall, the manuscript is well presented. I suggest the authors redraft the introduction to include a more comprehensive yet description of relevant research in the field (see recommendations below). More importantly is the issue of sample size selection, composition and justification (or lack thereof). I have some reservations about the joined analysis of exp 2 and 3 which I detail below.

***Introduction:***

- The comparison of biologically/socially relevant attentional cues (e.g eye-gaze, pointed hands, head orientation and body orientation) versus non-social cues (e.g arrows) has a rich history in the field of attention and more generally social cognition and perception. The introduction would benefit from a more in-depth description of some of the research in the field. Below are some references to relevant papers that the authors may wish to include. These are papers that have compared attentional orienting to social vs. non-social directional cues as well as papers that have examined perceptual representation of these directional cues. And more broadly, paper that have discuss how individual differences may account for the results we see in social attention with respect to cue type.

• Capozzi, F., & Ristic, J. (2018). How attention gates social interactions. Annals of the New York Academy of Sciences, 1426(1), 179-198.

• Dalmaso, M., Castelli, L., & Galfano, G. (2020). Social modulators of gaze-mediated orienting of attention: A review. Psychonomic Bulletin & Review,

27(5), 833-855.

• Guzzon, D., Brignani, D., Miniussi, C., & Marzi, C. A. (2010). Orienting of attention with eye and arrow cues and the effect of overtraining. Acta Psychologica, 134(3), 353-362.

• Cooney SM, O’Shea A, Brady N (2015) Point Me in the Right Direction: Same and Cross Category Visual Aftereffects to Directional Cues. PLoS ONE 10(10): e0141411.

*Response:* Thank you for your suggestions. We have now considered the studies you have mentioned for the revised version of the paper.

*Sample size, Composition & Statistical Power*

- How was the sample size estimated for the three experiments? 37 experiment 1, 75 experiment 2, 26 for experiment 3. Justification for sample size needs to be given.

*Response:* There was no experiment of reference for our first experiment, as this was the first time our paradigm was used. We could use as reference the study by Wiese et al., (2013), but they did not compare arrows and gaze, which was critical for our experiment. Instead, we could use Marotta et al. (2012) experiments, in which objects instead of group of objects (i.e., placeholders) were used, but they did compare gaze with arrow cues. Marotta et al. (2012) used samples of 24 and 30 participants, so we decided to use a sample of 36 (37 at the end) participants for Experiment 1. For experiment 2, we duplicated the sample size because of the online modality. We agree that in experiment 3, the sample was not very big. We decide to include this experiment because was basically a replication, and could be useful for performing a joint analysis with the placeholders data from Experiment 2. However, we agree that this experiment alone was not sufficiently powered and have decided to remove it from the revised version of the paper.

Unfortunately, as we did not have a clear reference experiment, we did not perform a priori power analysis. However, a sensitivity analysis carried out now for Experiment 1, with a sample size of 37 participants, with α = .05 and a power of 1-β=.90, showed that the experiment had enough sensitivity as to detect a minimum effect size of η^2^*_p_*=0.0202 (f= 0.143); the same analysis carried out for Experiment 2 (75 participants) showed that the experiment was sufficiently power as to detect a minimum effect size of η^2^*_p_*=0.0100 (f= 0.100). Importantly, these minimum effect size that could be detected by our design were smaller than the observed effect sizes for the critical interactions.

References

Marotta, A., Lupianez, J., Martella, D., & Casagrande, M. (2012). Eye gaze versus arrows as spatial cues: Two qualitatively different modes of attentional selection. *J Exp Psychol Hum Percept Perform*, *38*(2), 326-335. https://doi.org/2011-12265-001 [pii] 10.1037/a0023959

Wiese, E., Zwickel, J., & Müller, H. J. (2013). The importance of context information for the spatial specificity of gaze cueing. *Attention, Perception, &* *Psychophysics*, *75*(5), 967–982. doi: 10.3758/s13414-013-0444-y

- The majority of the sample in all three experiments is female. Please comment on this as a limitation with reference to previous research that has identified sex differences in spatial orienting of attention in similar Posner cueing experiments know differences in how males and female orient their attention (see Cooney, Brady & Ryan 2017; Bayliss, di Pellegrino &Tipper, 2005, Mitsuda, T., Otani, M., & Sugimoto, S. (2019).

*Response:* In fact, this is one of the main goals of a study we have now in preparation. We have already collected the full sample (female: 93, male: 90) and analysed the data. Results show that neither the interaction sex X cue-type (F_1,182_=0.421, p=.517), sex X validity (F_3,546_=0.644, p=.423), nor the sex X cue-type X validity (F_3,546_=1.086, p=.299) was significant. This lead us to the conclusion that no differences between males and females seem to be present in our study when using this task to dissociate between social and non-social attention.

- Exp 3 is a replication but with less than half the sample of experiment. The author’s then go on to run a combined analysis for experiment 2 & 3. Further justification is required here.

*Response:* Experiment 3 was added in order to replicate the findings. However, we understand that the sample size was notably smaller than Exp 2, the reason why following your advice, we have removed experiment 3 and the combined analysis from the revised version of the paper. Furthermore, we are carrying out one study (in preparation) in which we have used this task (placeholders present condition only) to compare social vs non-social attention in male and female students from STEM vs social sciences/humanities careers. Therefore, we are now confident that the observed pattern of data for the placeholders condition in Exp 2 replicates. Indeed, we replicated the Cue-Type x Validity, F_3,546_= 13.85, p<.001. Again, t-test analyses show that the general cueing effect was significant for both arrows (p<.001) and gaze (p=.015), but the object-based effect was only significant for arrows (p<.001), not for gaze (p=.322).

- Presumably, the combined analysis was conducted because Exp 3 contained a very small sample size. However, it’s important to note that the participants in Exp 3 only took part in one condition. While the authors mention this choice they do not justify it – why did the participants only do the placeholder present condition? The authors need to justify this choice in relation to the power of the sample in Exp 3. In general, the power and justification for sample size in all 3 experiments requires justification and discussion.

*Response:* We have removed Exp 3 and the combined analysis in the revised version of the paper.

- I have reservations about the combined analysis as it does not follow best research practice. Given that the result of the cue-type (arrow, eye gaze) * cue-target relation (same-location/same-hemifield vs. opposite-location/opposite- hemifield) changes (i.e. it becomes statistically significant) when the sample size is increased in the combined analysis and the study does not seem to be pre-registered, I recommend removing the combined analysis. Or at the very least detailing the choice and the possible limitations of this approach.

*Response:* We have removed Exp 3 and the combined analysis in the revised version of the paper.

Method

- For the arrow trials a horizontal line was placed… presumably this was a horizontal line with an arrowhead – this need to be specified.

*Response:* Thank you for the observation. This has been specified in the revised version of the paper (See Experiment 1, Method section: “Apparatus and stimuli”).

- Experiment 1 – Method: Please report the size and dimensions of the computer screen/monitor.

*Response:* Thank you for the observation. This has been mentioned in the revised version of the paper (See Experiment 1, Method section: “Apparatus and stimuli”).

- How was ‘unpredictability’ of the target X and O operationalized? Was there an equal amount of trials for X and O targets?

*Response:* Thank you for the observation. This has been specified in the revised version of the paper (See Experiment 1, Method section: “Procedure”).

- SOA: Would the authors expect different results if the SOA was manipulated? Why was 300ms chosen?

*Response:* The reason why an SOA of 300 ms was chosen was that recent meta-analytic evidence (Chacón-Candia et al., 2022 [under review]) has shown that SOAs of 200 to 400 ms report a stronger magnitude of gaze and arrow cueing effects (Figure 1), therefore, in order to shorten the duration of the experiment and because it is not relevant to the issues addressed in this article, we decided only to use an overall mean duration reported in previous evidence.

Figure 1

*Decrease of the standardized cueing effect with SOA. Filled circles represent studies using cues with long duration, whereas empty circles represent short cues (≤ 300 ms of duration). The size of the circles was proportional to the number of participants contributing to the effect. Dashed and dotted lines show the decrease in the cueing effects with long and short cue displays, respectively.*

References

Chacón-Candia J A, Román-Caballero R, Aranda-Martín B, Lupiáñez J, Casagrande M, Marotta A. (2022). No quantitative differences between eye-gaze and arrow cues: A meta-analytic answer and a call for qualitative differences. [Manuscript submitted for publication].

- Instead of describing three studies refer to each as Experiment 1, Experiment 2, Experiment 3.

*Response:* Thank you for the observation. This issue has been re-addressed in the revised version of the paper.

- Exp 2 and 3 are online experiments whereas Exp 1 was in-person. As Exp 2 & 3 were online, considerably less control over the way the stimuli were viewed, i.e did all participant take part on laptop/Pc’s?

*Response:* Yes, we made sure to make it sufficiently clear in the instructions given to participants that it was necessary to use a PC/laptop to successfully complete the task (responses to the target were to be made by pressing a key on the keyboard).

- Post-hocs should be reported for significant interactions. The authors refer to figures to make inferences about the interactions – at the very least the post- hocs should be in the supplementary.

*Response:* All relevant comparisons are now reported in the revised version of the paper.

- There are several grammatical and spelling errors.

*Response:* Thank you for the observation. The signalled errors have been corrected in the revised version of the paper. We also have reviewed the entire manuscript again to try to point out and correct any other possible errors.
