# Supplementary material for: Eye-Gaze direction triggers a more specific attentional orienting compared to arrows
Source: PLoS One. 2023 Jan 25;18(1):e0280955. doi: 10.1371/journal.pone.0280955 (PMC9876282; doi:10.1371/journal.pone.0280955)
Supplement: Attachment — Submitted filename: Response to Reviewers.docx [file pone.0280955.s003.docx]

October, 2022

Dear Academic Editor,

I hereby enclose the new revised version of manuscript number PONE-D-22-03696R1 titled *“Eye-Gaze direction triggers a more specific attentional orienting compared to arrows”.*

We very much appreciate your new comments as well as the reviewers’ new suggestions; we have tried to address all of them in this revised version of the manuscript. We agree that the points raised by you and by the reviewers continue to contribute to strengthen this article. Below we detail the changes in the manuscript in response to the latest comments made by you and the reviewers.

We hope that, after these latest changes the manuscript may now be suitable for publication. Moreover, we are willing to make any further changes if requested.

Thank you again for the new opportunity, and for your time and consideration.

Sincerely,

Jeanette A. Chacón-Candia (on behalf of the other authors)

Departamento de Psicología Experimental

Campus de Cartuja, s/n

18071 Granada – Spain

jeanettealicia.chaconcandia@uniroma1.it / chaconcandia@correo.ugr.es

**1. Editor**

Dear Dr. Chacón Candia,

Thank you for submitting your manuscript to PLOS ONE. After careful consideration, we feel that it has merit but does not fully meet PLOS ONE’s publication criteria as it currently stands. Therefore, we invite you to submit a revised version of the manuscript that addresses the points raised during the review process.

Thank you for replying to the reviewers’ comments. The MS has clearly benefited from these revisions and should have more widespread appeal given your consideration of the boarder literature on attentional cueing by socially relevant stimuli.

However, as editor, I have difficulties discerning whether you have addressed the concerns of both reviewers adequately, both of whom were chosen as experts in the area. Before considering the MS for publication in PLOS ONE, I would like to see the following issues addressed. The first is general, the others are specific to Reviewer 1 and 2 respectively

In many instances you simply reply to the reviewers’ concerns personally but do not incorporate these issues into the revised MS. While this may be appropriate for some concerns (e.g., if a reviewer raises an issue that is not part of your design it may be appropriate to communicate privately, as it were), in many cases the issues flagged by the reviewers are ones that readers will be interested in and have identical questions to the reviewers.

*Response:* Thank you for your comments. We have tried to address this general suggestion in the revised version of the paper.

***** As an obvious one, the shift from lab based to online work brings questions as to the size of the device a participant might use (PC screen, tablet, phone) which is potentially very important to grouping and proximity – while you reply to the reviewer you do not seem to have incorporated this into the revised MS.

*Response:* This issue has been explained more clearly in the revised version of the manuscript in the “Procedure” section of experiment 2 (p.15; lines 329-335 on revised MS).

Please provide a clean copy of the revised MS which:

**(a)** excludes removed material (i.e. all the crossed out text)

*Response:* We have considered this in the new version of the marked-up copy of the manuscript.

**(b)** uses track changes to highlight revised and new material

*Response:* We have considered this in the new version of the marked-up copy of the manuscript.

**(c)** please in the reply to reviewers indicate exactly where in the revised MS the changes have been made (pXX, lines XX-XX on revised MS).

*Response:* We have considered this in the new version of the marked-up copy of the manuscript.

**(d)** where you are replying privately to a reviewer and are NOT making changes to the MS, please say so explicitly

*Response:* We have considered this in the new version of the marked-up copy of the manuscript.

**Reviewer 1**

***** Reviewer 1 raises the important issue of how the statistical analysis was preformed, recommending for Exp 1 that you start with a repeated measures ANOVA with factors of cue-type (2 levels), placeholder-condition (2 levels) and validity (4 levels) to analyse the RT data. You have now done this but it very hard to see whether the ‘separate t-tests’ that you are refer to follow directly from this main ANOVA?

*Response:* Yes, t-tests are done following the interaction to analyse the general cueing and hemifield effects specifically. This has now been specified in the revised version of the paper in the "Design" section of experiment 1 (p.9; lines 202-204 on revised MS). We hope it is now clearer.

***** In presenting the results for this ANOVA you make no reference to cue-type – I assume this is because there is ‘not significant’ but as this is the crux of the research, it needs brief mention. The same issue arises for Exp 2

*Response:* Thank you for your observation; this has now been specified in the revised version of the paper in the “Results” section for both experiment 1 (p.12; lines 253-254 on revised MS) and experiment 2 (p.18; line 400-401 on revised MS).

**(a)** Please clarify that these analyses were post-hoc tests or planned comparisons arising from the main ANOVA.

*Response:* This is now specified in the new version of the paper (p.9; lines 202-204 on revised MS). T-tests are planned comparisons always comparing the two relevant conditions for our effects of interest: general cueing and hemifield/grouping effects. When we have no specific a priori hypotheses, we performed partial ANOVAs following the significant interactions. We hope this is now clearer in the manuscript.

***** Please submit an ANOVA table for Exp 1 and Exp 2. If you do not wish to include in the MS that is fine, but it would be helpful for the evaluation of the MS to have ANOVA results to hand

*Response:* Below you will find the complete tables of the results of the ANOVA analyses for experiments 1 and 2, respectively. We believe it is not necessary to include these tables in the manuscript.

**Experiment 1.**

**Experiment 2.**

**(b)** The language used is very confusing, e.g., by a ‘general cueing effect’ do you mean a cueing effect that is independent of the cue type (arrow/gaze)? If so, please state this.

*Response:* We are sorry for the confusion. The “general-cueing effect” refers to the comparisons between same-location/same-hemifield and opposite-location/opposite-hemifield conditions (p.9; line 204-206 on revised MS), and the hemifield-effect to that between same-hemifield and opposite-hemifield conditions (p.9; line 206-207 on revised MS). Depending on whether the validity effect is modulated by Cue type (Exp 2) or not (Exp1), t-tests for these effects are performed specifically for each cue-type or independently of it.

In other words, the general cueing effect is the standard effect observed when the target appears only in the placeholders located on each side of the cue. Although we understand the confusion with this term, we have used it following previous similar research (Marotta et al., 2012).

We hope this is clearer now in the manuscript.

Reference

Marotta, A., Lupiánez, J., Martella, D., & Casagrande, M. (2012). Eye gaze versus arrows as spatial cues: two qualitatively different modes of attentional selection. *Journal of Experimental Psychology: Human Perception and Performance*, *38*(2), 326. doi: 10.1037/a0023959.

- You seem to have taken the Reviewer’s advice as to how to analyse the data but are sticking with the terminology from the first MS?

*Response:* Indeed, we have followed his/her advice regarding the analyses. However, for analysing the interactions of interest (regarding the variable validity), we have kept the terminology general-cueing and hemifield/grouping effects as we believe these terms are helpful to differentiate distinct types of attentional orienting. Nevertheless, we hope that the revised version of the manuscript clarifies how these effects were analysed.

**(c)** It would be helpful if you would present the statistical results with direct reference to Figure 3 (for Exp 1) and Figure 6 (for Exp 2), and use an asterisk to denote significance in the plots where this occurs. Both these figures should have a visible and labelled y-axis

*Response:* Thank you for pointing this out. We have now included the references of figures 3 and 6 in the description of the results of experiment 1 (p.12; line 251 on revised MS) and experiment 2 (p.19; line 416-417 on revised MS) on the revised version of the manuscript. We also added asterisks to the figures to denote significance in the plots where this occurs, and we have labelled the y-axis.

**Reviewer 2**

***** Reviewer 2 highlights the essential issue of statistical power and sample size. For Exp 1 you justify your use of sample size with reference to published work. This is not good practice as much published work in experimental psychology is likely under-sampled.

*Response:* We have merged in one answer the critical points highlighted by Reviewer 2. You can find the complete response at the end of this comments section.

**(a)** Please provide a retrospective power analysis for Exp 1 as suggested by the reviewer, using conventional choices of effect size, power etc. As this is a repeated measures design you may wish to consider https://psycnet.apa.org/record/2019-45517-001

**(b)** Please address how your sample size compares with that recommended by the power analysis

**(c)** Please do the same for Exp 2. Here the sample size is considerable greater than for Exp 1 with no justification except for the ease of recruiting online.

**(d)** Although you have removed the formal comparison between Exp 1 and Exp 2 as recommended by the reviewer, you still need to consider the implications of different sample sizes for your conclusions, e.g., is it appropriate to conclude that gaze acts differently than arrows but only in the case of grouped stimuli?. Because you do see a 3-way interaction in Exp 2 but not in Exp 1? Is it possible that the difference in the results come down to sample size?.

Given the importance of power analyses, sample size etc and the considerable issues of moving from laboratory based to online research across the two experiments, it may be wise to discuss with your research team conducting Exp 2 in the lab with formal power analysis?

*Response:* We agree with the reviewer that it is not good practice to base sample size estimation on previous estimations. Indeed, we currently conduct a priori power analysis to estimate the needed sample size for our studies. However, the data from the experiments reported in the current paper were collected before this practice was fully established in our lab. As far as we know, the best is rather to report sensitivity analysis, showing the minimum effect size that could be detected with our sample size. Therefore, we now report sensitivity analyses for both Experiments 1 and 2 (p's.6 and 7; lines 139-143 and p.14; lines 309-313, respectively, on revised MS).

Of course, we agree that replicating in the lab with an a priori estimated sample size would be ideal. However, we have already replicated online the same pattern of data observed in Experiment 2 (placeholders present condition), in which we investigated in a large sample size of 200 men and women students from different careers (STEM vs social sciences) the effects observed for arrows and gaze. Although no differences between gender or sex were observed, the overall pattern was replicated: whereas arrows led to both general-cueing and grouping effects gaze only produced a significant general-cueing effect. We are therefore confident that the data pattern is reliable and replicable online.

**Minor issues**

1. With regard to reference 28 (Chacón-Candia J A, Román-Caballero R, Aranda-Martín B, Lupiáñez J, Casagrande M, Marotta A. please use the standard method of citing online publications and do not use “Manuscript submitted for publication”: You can find the correct citation at <https://psyarxiv.com/>

*Response:* Thank you for pointing this out. The citation has been corrected in the revised version of the manuscript (p.28; lines 632-635 on revised MS).

2. Table 1 You refer to percent error yet use abbreviation IR (I assume this is ‘incorrect response’?). Please use either one or the other as it is confusing when the abbreviation does not match the long text.

*Response:* Thank you for your observation; this issue has been corrected in both table 1 (p.10; line 219 on revised MS) and table 2 (p.17; lines: 378 on revised MS) of the revised version of the manuscript.
